# Mitochondrial Genome Instability in *W303-SK1* Yeast Cytoplasmic Hybrids

**DOI:** 10.3390/biology13110927

**Published:** 2024-11-14

**Authors:** Khoren K. Epremyan, Arteom A. Burlaka, Olga V. Markova, Kseniia V. Galkina, Dmitry A. Knorre

**Affiliations:** 1 A.N. Belozersky Institute of Physico-Chemical Biology, Lomonosov Moscow State University, Leninskiye Gory, 1-40, 119234 Moscow, Russia; k.epremyan19@gmail.com (K.K.E.); markova@belozersky.msu.ru (O.V.M.); galkinakseniia@gmail.com (K.V.G.); 2 Faculty of Bioengineering and Bioinformatics, Lomonosov Moscow State University, Leninskiye Gory, 1-73, 119234 Moscow, Russia; zok.miodov.27@gmail.com

**Keywords:** yeast, mtDNA, cytoplasmic hybrids, suppressivity, mito-nuclear incompatibility

## Abstract

Unlike most animals, some fungi, including baker’s yeast, inherit mitochondrial DNA (mtDNA) from both parents. When haploid yeast cells fuse, they form a heteroplasmic zygote, whose offspring retain one or the other variant of mtDNA. Meanwhile, some mutant mtDNA (*rho^−^*), with large deletions in the nucleotide sequence, can displace wild-type (*rho^+^*) mtDNA. Consequently, offspring of zygotes with such *rho^−^* mtDNA predominantly carry the mutant variant. This phenomenon is called suppressivity. In this study, we investigated how the suppressivity of *rho^−^* mtDNA depends on the mitochondrial and nuclear genomes of the *rho^+^* strain during crossing. Comparing two diverged laboratory strains, *SK1* and *W303*, we measured suppressivity in crosses with four *rho^−^* strains. One *rho^−^* strain showed significantly higher suppressivity when crossed with *SK1* than with *W303*. We then created cytoplasmic hybrids by swapping mtDNAs between these strains. Surprisingly, we found that the mtDNA of the *rho^+^* strain, rather than its nuclear DNA, determines high suppressivity in crosses of *SK1 rho^+^* with the *rho^−^* strain. Additionally, mtDNA replacement reduced respiration rate and growth rate on non-fermentable substrates while increasing the likelihood of functional mtDNA loss. Our data demonstrate that a mutant mtDNA variant’s ability to displace another mitochondrial DNA variant in a heteroplasmic cell depends more on mtDNA sequences than on the biochemical and structural context created by the nuclear genome background.

## Simple Summary

Mitochondrial respiratory chain subunits are encoded by both mitochondrial and nuclear genomes, imposing evolutionary constraints due to potential incompatibilities between mutations in these genomes. Our study demonstrates that swapping mitochondrial DNA (mtDNA) between two laboratory yeast strains increases the frequency of mtDNA loss and reduces their respiratory capacity, suggesting coadaptation of mitochondrial and nuclear genomes at the intraspecific level. We also show that the ability of selfish mtDNA to displace other variants in heteroplasmic yeast cells depends primarily on their mtDNA sequences rather than the nuclear genome background. These findings underscore the complex interactions between mitochondrial and nuclear genomes.

## 1. Introduction

Mitochondria of most eukaryotic species harbor their own DNA, with very few exceptions [1]. Meanwhile, the life cycle of many fungi involves the fusion of cytoplasms, which predates the fusion of nuclei [2]. As a result, a single cell can harbor mitochondria with diverged mitochondrial DNAs (mtDNAs); this condition is usually referred to as mitochondrial heteroplasmy. As a result of genetic drift, the descendants of heteroplasmic cells eventually retain only one of the mtDNA variants. In baker’s yeast, such segregation takes only a few cell divisions, with the exact number depending on parameters such as the structure of the mitochondrial cristae and the nature of substitution in mtDNA [3,4].

Laboratory yeast strains have high frequencies of mutations in mtDNA, which results in a high proportion, up to several percent, of petite yeast cells in suspensions of yeast cells [5]. Petite cells are incapable of oxidative phosphorylation and cannot utilize non-fermentable carbon sources, such as glycerol or ethanol [6]. The mtDNA of such cells usually harbors large deletions and is devoid of mitochondrially encoded protein-coding genes, tRNAs, and rRNAs (referred to as *rho^−^* mtDNA) [7]. Despite this, such mutant mtDNAs can have a fitness advantage at the intracellular level over wild-type *rho^+^* mtDNAs [8,9]. Therefore, during mating, after fusion of the yeast gametes, such *rho^−^* mtDNAs can displace wild-type *rho^+^* mtDNAs. As a result, the crossing of some *rho^−^* haploid strains with a *rho^+^* strain of opposing mating type could produce up to 100% *rho^−^* diploids [10,11]. This phenomenon is called suppressivity and is usually defined as the proportion of *rho^−^* diploids among all diploids produced in *rho^−^* × *rho^+^* strain crossings.

The mechanisms of suppressivity are still unclear. On the one hand, *rho^−^* mutant mtDNAs with large deletions still contain highly active origins of replication and, therefore, their replication takes less time than the replication of full-length *rho^+^* mitochondrial DNA [12], although the increased rate of *rho^−^* mtDNA replication is observed only in some *rho^−^* strains [10]. Similarly, the deficiency of deoxynucleotides, which hampers mitochondrial DNA replication, can also provide a replication rate advantage to the *rho^−^* mtDNAs [13].

On the other hand, it has been shown that several mitochondrial enzymes are required for the suppression of wild-type mtDNA by *rho^−^* mtDNA. For example, mitochondrial RNA polymerase Rpo41p unexpectedly impaired the maintenance of *rho^+^* mtDNAs in heteroplasmic zygote cells, while the reduction in its activity decreased the suppressiveness of *rho^−^* strains [14]. The activity of Rpo41 is regulated by mitochondrial RNA endonuclease Pet127p; however, the regulation mechanism is not associated with the RNase activity of Pet127p but rather with the physical interaction between Rpo41p and Pet127p [14]. Moreover, genetic screening revealed that the deletion of the *CCE1* gene (alias *MGT1*) abolished the suppressiveness of highly suppressive *rho^−^* strains [15]. *CCE1* encodes the cruciform cutting endonuclease which is required for mitochondrial recombination [16]. In contrast to mammalian mtDNA [17], yeast S. cerevisiae mtDNA experiences frequent recombinations [18]. Moreover, *rho^−^* mtDNAs can recombine with *rho^+^* mtDNAs. This observation formed the basis of another hypothesis about the mechanisms of suppressivity. According to this suggestion, mutant *rho^−^* mtDNA can enter into destructive recombination with *rho^+^* mtDNA [19]. Considering the high copy number of *rho^−^* mtDNA in the cells, this destructive recombination process removes most *rho^+^* mtDNA molecules from the heteroplasmic cells, resulting in a transmission bias favoring *rho^−^* mtDNAs.

Although there are multiple possible mechanisms that confer suppressivity of *rho^−^* mtDNAs, they can be classified into two distinct categories. First, some mechanisms should depend on mtDNA genotypes. For example, if the main contribution to suppressivity is made by the relative activity of the origins of replication of *rho^−^* and *rho^+^* molecules, or if the consequences of destructive recombination are determined by the homology of mtDNA sequences, then suppressivity will depend on the mtDNA genotype. Second, suppressivity may depend on the presence of certain alleles in nuclear DNA (nDNA), for example, variants of *CCE1*, *RPO41*, *PET127*, or other yet unidentified nuclear-encoded genes regulating suppressivity.

In the study presented herein, we tested the contribution of these mechanisms by measuring the suppressivity of several previously characterized *rho^−^* strains by crossing them with two diverged standard laboratory strains, *SK1* and *W303-1A*, available in our laboratory. We found that one of the *rho^−^* strains exhibited significantly different suppressivity while crossing with *SK1 rho^+^* and *W303-1A rho^+^* strains. Thus, we produced cytoplasmic hybrids by swapping the mtDNA of the *SK1* and *W303* strains and found that the differences in their mtDNA, rather than their nDNA, explain this variation.

## 2. Materials and Methods

### 2.1. Yeast Strains and Growth Conditions

This study utilized *Saccharomyces cerevisiae* strains *W303-1A*, *SK1*, and *NAB69*, along with their derivative mutants (Table S1). Yeast strains were cultured in standard rich and synthetic media as described by Sherman [20]. The rich medium components included peptone (DiaM (Moscow, Russia), Cat. No. HYP-A.5000) and yeast extract (BioSpringer (Cedar Rapids, IA, USA), Cat. No. 0207/0-PW-L.5000). Agar (DiaM (Moscow, Russia), Cat. No. 1923.5000) was used for solid media preparation. Carbon sources included D-glucose (Helicon (Moscow, Russia), Cat. No. H-0401-0.5), raffinose (Chimmed (Moscow, Russia), Cat. No 2012), and glycerol (Helicon (Moscow, Russia), Cat. No. I-195204-1.0). Carbon sources were autoclaved separately from other components. The medium’s pH was approximately 5.5. Synthetic media were prepared using Yeast Nitrogen Base without amino acids (Sigma (San Diego, CA, USA), Cat. No. Y0626-1KG) and yeast synthetic drop-out medium supplements (Sigma (San Diego, CA, USA). When required, G418 sulfate (PhytoTechnology Laboratories (Lenexa, KS, USA), Cat. No. 108321-42-2) was added for selection.

### 2.2. Comparison of SK1 and W303 Genomes

**Figure 1:**
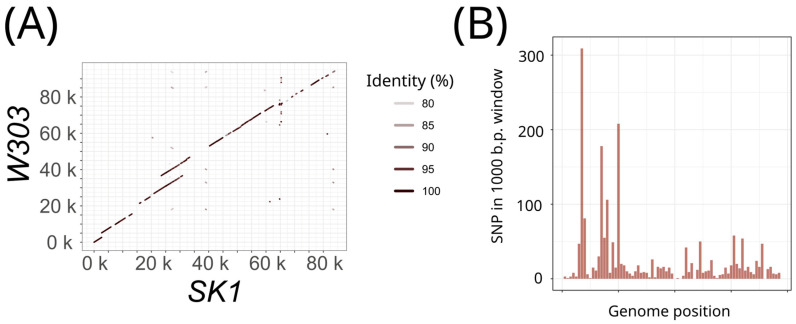
Divergence of *SK1* and *W303* mitochondrial genomes. (**A**) Local similarity plot comparing *SK1* and *W303* mitochondrial genomes; (**B**) nucleotide substitutions in the aligned mitochondrial genomes of *SK1* and *W303* strains (see Section 2).

**Figure 2:**
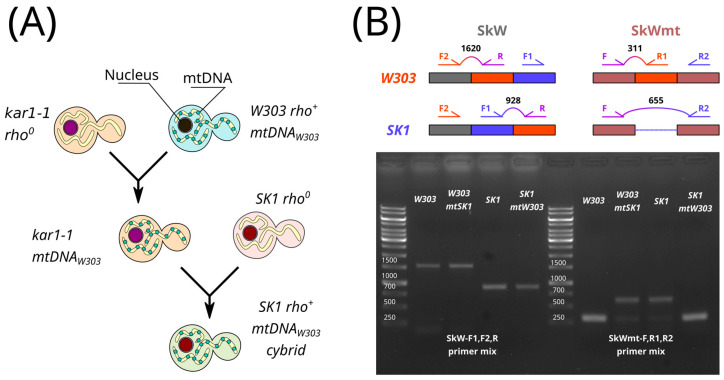
Replacement of mtDNA in *SK1* and *W303* strains. (**A**) Schematic of *SK1mtW303* cybrid production. (**B**) PCR verification of mtDNA replacement in *W303mtSK1* and *SK1mtW303* cybrids. The scheme represents genomic sites exhibiting differences in *W303* and *SK1* mitochondrial (**right**) and nuclear (**left**) genomes. Positions of primers (see Tables S2 and S3) and expected PCR product lengths are shown. The gel image below displays PCR products obtained using genomic DNA from parental strains or cybrids and primer mixes SkW (nDNA) or SkWmt (mtDNA).

To distinguish the nuclear and mitochondrial genomes of *SK1* and *W303* (see Table S3) strains by PCR and following agarose gel electrophoresis, we found loci providing products of different sizes with the same primers. For that purpose, we made local similarity graphs (Figure 1A and Figure S1) using the “blastn” program [21] available at the NCBI. This showed us rearranged loci, and we designed the primers to produce significantly different products (see Figure 2).

To count SNPs in mitochondrial genomes, we performed global alignment with PairwiseAligner (scoring = ‘blastn’) from biopython [https://biopython.org/, Current Release—1.84—28 June 2024], removed all columns with gaps (Figure S2), and calculated SNPs in 1000 b.p. windows without overlaps.

### 2.3. Yeast Cybrid Strain Construction and Confirmation

To construct the cybrids, we utilized two rounds of crossing with the *rho^0^* strains (see the scheme in Figure 2A). The *NAB69 Δkar1-1 rho^0^* strain was then crossed with *W303 KanMX6 rho^+^* and *SK1 KanMX6 rho^+^* strains. We preincubated the cells in liquid YPD medium at 30 °C for at least 4 h. Crosses were performed by mixing the cells to an OD_550_ of 0.1 in 200 μL of YPD and incubating them for 20 h at room temperature. The resulting suspension was diluted 100-fold and plated on solid YNBGly -Leu dropout medium. The plates were incubated for 48–72 h at 30 °C. Colonies were subsequently transferred to both YNBGly-Leu and YNBGly-Leu G418 plates to select for haploid cybrids containing the *NAB69 Δkar1-1* nucleus and mitochondrial DNA from either *W303* or *SK1*. To validate the resulting cybrids, they were crossed with *SK1 TRP rho^0^* and *W303 TRP rho^0^* strains.

The cybrids were confirmed by polymerase chain reactions (PCRs) with the primers designed to anneal unique regions of either *SK1* or *W303* mitochondrial and nuclear DNA (Tables S2 and S3). The PCRs were carried out using Phusion Hot Start II high-fidelity DNA polymerase. The PCR mixtures contained Phusion HF buffer, 200 μM dNTPs, the corresponding primers (at a final concentration of 0.5 μM), and yeast cells as the DNA template.

### 2.4. Suppressivity Test (rho^−^ mtDNA)

The cells were incubated in liquid YPD medium at 30 °C for at least 4 h. MAT a *rho^+^* strains were then crossed with either MAT alpha *rho^−^* strains or control MAT alpha *rho^+^* and *rho^0^* strains. Diploid selection was achieved using prototrophic selective markers (*URA3* and *TRP1*). For crossing, the cells were mixed to an OD_550_ of 0.1 in 200 μL of YPD and incubated for 20 h at room temperature. The resulting suspensions were diluted 100–1000-fold in sterile water and plated on synthetic dropout DGly (0.1% d-glucose, 2% glycerol) medium without uracil and tryptophan (-Ura, -Trp). The plates were incubated at 30 °C for 48–72 h, after which colonies were counted. Suppressivity was calculated as (number of petite CFUs/total CFUs) × 100% on YPDGly.

### 2.5. Growth Kinetics

We incubated the strains in liquid YPGly medium at 30 °C for at least 4 h. Exponentially growing cells were then diluted to an OD_550_ of 0.05 and inoculated into a 96-well plate (Eppendorf (Hamburg, Germany). The plates were incubated at 30 °C in a SpectrostarNANO (BMG Labtech (Ortenberg, Germany) spectrophotometer with orbital shaking at 500 rpm for 2 min prior to each measurement. Optical density was measured at 5 min intervals over a period of 20–27 h. The maximal growth rate (μ_max_), which is proportional to the inverse of duplication time, was calculated using linear model fits of the natural log-transformed OD_550_ values. This analysis was performed using an R script as described in [22]. Along with μ_max_, the script provides R^2^ values that reflect the quality of the linear fit. We noticed that *SK1* strain cells could aggregate and, therefore, in some experiments, the data were noisy, thus providing lower R^2^ values.

### 2.6. Respirometry

We assessed the respiration of cells with a Clark-type oxygen electrode (Strathkelvin Instruments 782, Scotland, UK) at 25 °C. The incubation medium was 50 mM KH_2_PO_4_ and 0.05% glucose, pH = 5.5.

### 2.7. Statistics, Data Visualization, and Analysis

All experiments were performed with at least five independent replicates. The figures were made using the tidyverse packages of the R language [23]. The figures show boxplots where the lower and upper borders correspond to the 25th and 75th percentiles (InterQuartile Range, IQR). The upper whisker extends from the box border to the largest value within 1.5 × IQR. The lower whisker reaches the smallest value within 1.5 × IQR. The results of individual data points are shown as circles; points beyond whiskers are outliers. All analyses were performed using the base R programming language library. The significance difference was determined using the Wilcoxon rank-sum exact test. We plotted only the significance of the comparison with *p*-values below 0.05.

## 3. Results

To assess whether the mitochondrial or nuclear genome of the *rho^+^* strains determines the suppressivity of the *rho^−^* strains, we selected two laboratory strains, *SK1* and *W303*, which harbored the required set of genetic markers for testing suppressivity. Importantly, the mitochondrial genomes of these strains exhibited pronounced differences in architecture and accumulated numerous single nucleotide polymorphisms (SNPs) (Figure 1A,B). Figure 1A displays a local similarity plot highlighting deleted and duplicated mtDNA regions in the *W303* mitogenome compared to that of *SK1*. Figure 1B shows the number of SNPs in a 1000 bp sliding window along the genome alignments. These results demonstrate that the mitochondrial genomes of *SK1* and *W303* have diverged sufficiently to be distinguishable, while potentially inhibiting recombination between them. Meanwhile, the nuclear genomes of *SK1* and *W303* strains also show some differences in architecture, including duplications and rearrangements (see, for example, the local similarity graph of chromosome I in Figure S1). The genes *CCE1*, *RPO41*, and *PET127*, which affect mitochondrial DNA suppressivity, differ by only 2 to 4 missense non-radical substitutions in the variable regions of their orthologs in other Ascomycota species (Supplementary Text S1).

We then produced two cytoplasmic hybrids (cybrids) using a *kar1-1* strain as an intermediate host for the mtDNA. The *kar1-1* mutation inhibits nuclear fusion, often resulting in dikaryotic cells that enable the transduction of *rho^+^* mtDNA into a new nuclear background (Figure 2A). Using this approach, we swapped mtDNA between *W303* and *SK1* strains, generating a *W303* cybrid strain with *SK1* mtDNA (*W303mtSK1*) and an *SK1* cybrid strain with *W303* mtDNA (*SK1mtW303*). To confirm the presence of the correct nuclear and mitochondrial DNAs in these cybrids, we performed colony PCR using strain-specific primers (Table S2). The primers used to verify mitochondrial replacement in the cybrids yielded PCR products of varying sizes for the *SK1* and *W303* genomes, as expected (Figure 2B).

**Figure 3:**
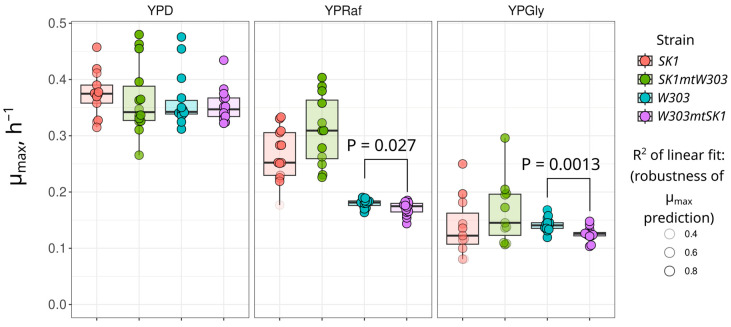
Growth rates of *W303mtSK1* and *SK1W303* cybrids in rich medium with fermentable (YPD), poorly fermentable (YPRaf), and non-fermentable (YPGly) carbon sources. Data points represent individual experiment growth rates. Color opacity indicates μ_max_ calculation fit quality (see Section 2). Statistical significance was determined using the Wilcoxon rank-sum exact test.

**Figure 4:**
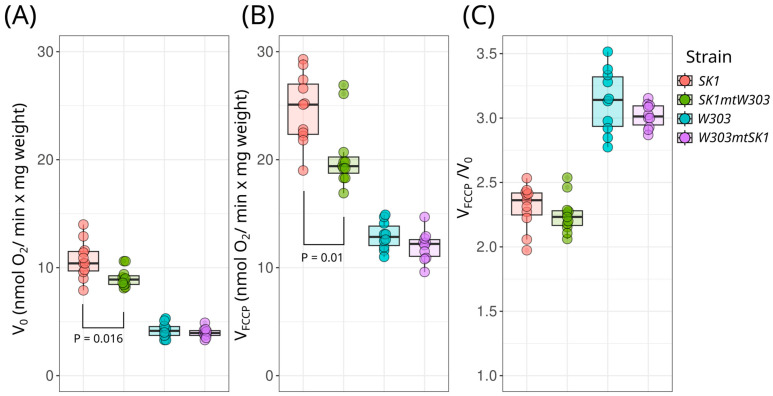
Respiration rates of intact *W303mtSK1* and *SK1mtW303* cybrid cells. (**A**) Basal respiration rate (V_0_); (**B**) maximal respiration rate in the presence of 5 μM FCCP protonophore (V_FCCP_); (**C**) respiratory control ratio (V_FCCP_/V_0_), reflecting mitochondrial coupling efficiency. Statistical significance was determined using the Wilcoxon rank-sum exact test.

To characterize the phenotypes of the cybrids, we measured the specific maximal growth rates of the cybrids and parental strains. We tested their growth in rich media with different carbon sources: fermentable (YPD), poorly fermentable (YPRaf), and non-fermentable (YPGly). While no difference was detected in YPD, the *W303mtSK1* cybrid exhibited slower growth in YPRaf and YPGly (Figure 3). Given that mitochondrial DNA encodes respiratory chain components, we also measured the respiration rate of the cybrids and their parental strains. Figure 4 shows that the oxygen consumption rate of *SK1mtW303* cybrid cells was lower than that of the parental *SK1* strain. This effect was pronounced in both tested conditions: with and without the addition of the protonophore Carbonyl cyanide 4-(trifluoromethoxy) phenylhydrazone (FCCP). FCCP uncouples respiration and phosphorylation, thus enabling maximal activity of the respiratory chain. The *W303mtSK1* cybrid exhibited a slower decrease in respiration rate compared to the parental *W303* strain, although this difference was not statistically significant.

**Figure 5:**
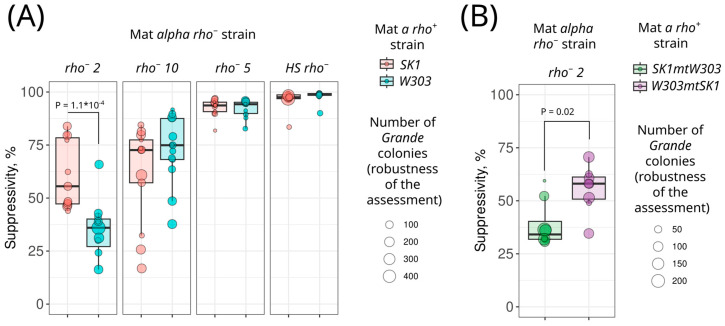
Strain-specific inheritance bias (suppressivity) of *rho^−^* mitochondrial DNA. (**A**) Suppressivity of four *rho^−^* strains crossed with *W303* and *SK1 rho^+^* strains; (**B**) suppressivity of cytoplasmic hybrids crossed with *rho^−^ 2*. Statistical significance was determined using the Wilcoxon rank-sum exact test. The size of the individual data points represents the sample size of the individual experiments, which is the total number of colonies on the agar plate.

The premise of this study was the hypothesis that the suppressivity of *rho^−^* strains can differ depending on the *rho^+^* strain with which it is crossed. To test this, we selected four previously characterized MAT alpha *rho^−^* strains with varying suppressivity [9] and crossed them with two MAT a strains, *SK1* and *W303*. Our results showed that for highly suppressive strains (HS *rho^−^*, *rho^−^* 5, and *rho^−^* 10), substitution of the *rho^+^* strain did not significantly affect suppressivity (Figure 5A). However, the low-suppressive strain *rho^−^* 2 produced significantly more petite colonies when crossed with the *SK1 rho^+^* strain compared to the *W303 rho^+^* strain (Figure 5A). Notably, control crossings of the *SK1* strain with *rho^0^* and *rho^+^* strains produced no petite colonies. Interestingly, the *rho^−^* 2 strain exhibited lower suppressivity when crossed with *SK1mtW303* than when crossed with the *W303mtSK1* cybrid (Figure 5B). This result indicates that the mitochondrial DNA, rather than the nuclear DNA background of the *rho^+^* strain, explains the variation in suppressivity values.

**Figure 6:**
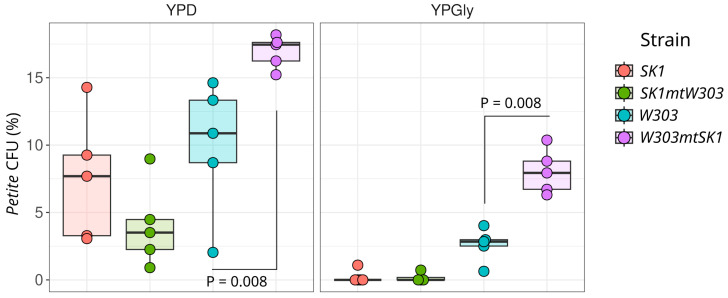
Increased mitochondrial genome instability in the *W303* strain after mtDNA replacement. The cells were cultured in YPD and YPGly media and then plated on YPDGly. The graph shows the percentage of petite colony-forming units (CFUs). Statistical significance determined using the Wilcoxon rank-sum exact test.

We hypothesized that the decreased resistance of the *W303mtSK1* strain to *rho^−^* 2 mtDNA is due to incompatibility between the *W303* nuclear background and *SK1* mtDNA. To test this, we measured the percentage of spontaneous petite cells in the *W303mtSK1* suspension grown in YPD and YPGly media. In YPD medium, emerging *petite* cells can continue to divide, albeit at a slower rate than the parental *rho^+^* strain. In contrast, YPGly medium contains only a non-fermentable carbon source, allowing only *rho^+^* cells to proliferate. As expected, we found that all tested strains contained more *petite* cells when grown on YPD compared to YPGly medium (Figure 6). Importantly, on both tested media, the *W303mtSK1* strain produced significantly more petite cells than the parental *W303* strain. This observation suggests that the mitochondrial DNA of the *SK1* strain cannot be stably maintained in the *W303* background. Meanwhile, the complementary *SK1mtW303* strain did not show mitochondrial genome instability.

## 4. Discussion

mtDNA encodes core subunits of the respiratory chain complexes and ATPase, while other subunits of these complexes are encoded in nuclear DNA. This arrangement imposes restrictions on the evolution of genes encoding these complexes, as mutations in one genome may become incompatible with the other [24,25,26]. Consequently, replacing mtDNA is only possible between closely related species. For instance, replacing human mtDNA with mtDNA from chimpanzees and gorillas produces cells capable of oxidative phosphorylation (OXPHOS). However, cytoplasmic hybrids of humans and orangutans are not viable [27]. Interestingly, the speciation of some organisms, including yeast, is driven by mitochondria–nucleus incompatibility [28,29].

Yeast cells have proven to be a valuable model for studying mito-nuclear interactions due to their ability to proliferate without mitochondrial DNA, which expands the available methods for manipulating their mitochondrial genomes. In this study, we swapped mtDNA and nDNA between two standard laboratory haploid strains: *SK1* and *W303*. The *W303* strain is one of the most commonly used laboratory strains [30]. Notably, the *rho^−^* strains used in this study were derived from the isogenic *W303* MAT alpha strain [9,11], and are therefore expected to have almost identical nuclear genomes with only minor MAT locus rearrangements in the nDNA. The *SK1* strain is frequently employed in meiosis studies due to its high sporulation efficiency [31]. The genomes of these strains are pronouncedly diverged compared to other standard laboratory strains [32,33].

As expected, non-native mtDNA decreased the performance of yeast strains under respiratory conditions. We found that *W303mtSK1* cybrids exhibited a decreased growth rate on non-fermentable and poorly fermentable carbon sources (Figure 3). Since oxidative phosphorylation is required for efficient utilization of these carbon sources, we suggest that even mild mito-nuclear incompatibility between *SK1* and *W303* mtDNA led to a decrease in fitness. However, we did not observe a difference in growth rate between parental *W303* and *W303mtSK1* strains in the YPD medium (Figure 3). This can be explained by the fact that under glycolytic conditions, mitochondrial functions are suppressed [34] and enzyme complexes containing proteins encoded by mtDNA do not play a significant role.

Meanwhile, we found that the *SK1mtW303* cybrid exhibited a lower respiration rate compared to the parental *SK1* strain (Figure 4). The *W303mtSK1* strain also showed a decrease in respiration rate compared to its parental strain, although this difference was not statistically significant. It remains unclear why the decrease in respiration rate of the *SK1mtW303* strain did not manifest as a growth defect when using respiratory substrates. We hypothesize that the *SK1* strain’s growth rate may be additionally limited by other factors, such as its ability to flocculate, which could mask growth defects in the YPGly medium

The variation in individual yeast strain phenotypes is primarily explained by nDNA sequence and mito-nuclear interactions, while mtDNA sequence itself poorly explains phenotypic variation [35,36]. Our study demonstrates that mtDNA sequence can, in some cases, have a major effect on phenotype under conditions of mitochondrial heteroplasmy. We showed that mitochondrial DNA, rather than nuclear DNA, can determine *rho^+^* mtDNA susceptibility to suppression by *rho^−^* mtDNA (Figure 5). Specifically, the low-suppressive mtDNA, *rho^−^ 2*, displaced non-related *SK1* mtDNA in the suppressivity test more efficiently than parental *W303* mtDNA (Figure 5). Interestingly, we did not observe any difference in suppressivity for the other tested *rho^−^* strains depending on the *rho^+^* strain with which they were crossed. This may be explained by different mechanisms of suppressivity in highly and low-suppressive *rho^−^* mtDNAs. Alternatively, in the case of the highly suppressive *rho^−^* strain, the sensitivity of the method might not be sufficient to quantify the difference. Indeed, in the *HS rho^−^* crosses, less than one percent of *Grande* colonies that were able to retain wild-type mtDNA formed (Figure 5).

## 5. Conclusions

To summarize, our study demonstrates that swapping mtDNA between laboratory yeast strains increases the rate of mtDNA loss while decreasing respiration and growth rates. Although expected, this observation suggests that mutual adaptation of mitochondrial and nuclear genomes occurs at the intraspecific level in yeast. At the same time, our data show that the mutant mtDNA variant’s ability to outcompete another variant in a heteroplasmic cell can be primarily determined by mtDNA sequences rather than the nuclear genome background.

In the state of mitochondrial heteroplasmy, over the course of generations, only one variant of mitochondrial DNA usually remains in the cells. This outcome is determined by both random genetic drift and the relative fitness levels of the different mtDNA variants [37]. Some variants have been shown to reproducibly displace others. The relative fitness of different variants depends on the context (e.g., tissue type of the multicellular organism), which is created in the mitochondrial matrix by proteins encoded in the nuclear genome [38,39]. However, the results of suppressivity tests in yeast *SK1-W303* cybrids demonstrate that, in some cases, the relative competition of mtDNA in yeast is determined by differences in the mtDNA itself. These results demonstrate that different mtDNAs from standard laboratory strains can be subject to varying degrees of displacement by selfish mitochondrial DNA elements. We propose that mtDNA sequences evolved under constant pressure from such selfish elements and may contain adaptations to counteract them.

## Data Availability

The data used to support the findings of this study are available from the corresponding author upon request.

## Supplementary Materials

The following supporting information can be downloaded at: https://www.mdpi.com/article/10.3390/biology13110927/s1, Figure S1: Local similarity graph for chromosome 1 of *SK1* and *W303* strains. The rearrangement used for verifying PCR is shown at approximately 160,000 base pairs (160 kb) along the genomic coordinates; Figure S2. Gaps in the alignment of *SK1* and *W303* mitochondrial DNA. This figure shows the gaps in the alignment of *SK1* and *W303* mtDNA that were excluded from the analysis presented in Figure 1B. Blue bands represent columns with gaps. Both sequences start at the *CO1* gene; Table S1: List of strains used in this study; Table S2. List of primers; Table S3. Primer binding sites coordinates in sequence files. References [9,11,40] are cited in the supplementary materials.
