# Supplementary material for: Mitochondrial Genome Instability in W303-SK1 Yeast Cytoplasmic Hybrids
Source: Biology (Basel). 2024 Nov 14;13(11):927. doi: 10.3390/biology13110927 (PMC11592453; doi:10.3390/biology13110927)
Supplement: Supplementary file 1 [file biology-13-00927-s001.zip › biology-3287681-supplementary.pdf]

Supplementary materials: Mitochondrial genome instability in *W303-SK1* yeast cytoplasmic cybrids

Khoren K. Epremyan[1], Arteom A. Burlaka[2], Olga V. Markova[1], Kseniia V. Galkina[1], Dmitry A. Knorre[1, *]

[1]A.N. Belozersky Institute of Physico-Chemical Biology, Lomonosov Moscow State University, 119234, Moscow, Leninskiye Gory, 1-40

[2]Faculty of Bioengineering and Bioinformatics, Lomonosov Moscow State University, 119234, Moscow, Leninskiye Gory, 1-73

* knorre@belozersky.msu.ru

Table S1. List of strains used in this study.

Strain	Genotype	Parental strains and/or references
W303 URA3[b]	*MATalpha, URA3, rho*$^+$	*W303-1A* from Laboratory of A.Hyman
W303 URA3 rho^0 [a]	*MATalpha, URA3, rho*^{0}	*W303 URA3*
W303 TRP1[b]	*MATa, TRP1, rho*$^+$	*W303-1A* from Laboratory of A.Hyman
W303 TRP1 rho^0 [a]	*MATa, TRP1, rho*^{0}	*W303 TRP1*
W303 KanMX6[b]	*MATa, HSP104-GFP::KanMX6, rho*$^+$	*W303-1A* from Laboratory of A.Hyman

W303 2 rho^{-}	*MATalpha, URA3, rho*$^{-}$	*(Kashko et al. 2024)*
W303 HS rho^{-}	*MATalpha, URA3, rho*$^{-}$	*(Karavaeva et al. 2017)*
W303 5 rho^{-}	*MATalpha, URA3, rho*$^{-}$	*(Kashko et al. 2024)*
W303 10 rho^{-}	*MATalpha, URA3, rho*$^{-}$	*(Kashko et al. 2024)*
SK1 TRP1[b]	*MATa, TRP1, rho*$^{+}$	*SK1 presented by S. Dmitriev*
SK1 TRP1 rho^{0} [a]	*MATa, TRP1, rho*^{0}	*SK1 TRP1*
SK1 KanMX6[b]	*MATa, mca1::KanMX6, G418, rho*$^{+}$	*SK1 presented by S. Dmitriev*
NAB69 Δkar1-1	*MATalpha, LEU2, rho*^{0}*, Δkar1-1*	*(Conde and Fink 1976)*
NAB69 Δkar1-1 mtW303 cybrid [c]	*MATalpha, LEU2, rho*$^{+\ W303}$*, Δkar1-1*	*NAB69 Δkar1-1, W303 G418*
NAB69 Δkar1-1 mtSK1 cybrid [c]	*MATalpha, LEU2, rho*$^{+\ SK1}$*, Δkar1-1*	*NAB69 Δkar1-1, SK1 G418*
W303 mtSK1 cybrid [c]	*MATa, TRP1, rho*$^{+\ SK1}$	*W303 TRP1 rho*^{0}*, NAB69 Δkar1-1 mtSK1 cybrid*
SK1 mtW303 cybrid [c]	*MATa, TRP1, rho*$^{+\ W303}$	*SK1 TRP1 rho*^{0}*, NAB69 Δkar1-1 mtW303 cybrid*

[a]Strain produced by ethidium bromide-induced loss of mitochondrial DNA.

[b]Strain produced by the transformation of the PCR cassette.

[c]Cybrid produced by mating with *NAB69 Δkar1-1* strain.

Table S2. List of primers.

Name	Sequence 5’-3’
SkWmt-F	CCGTATGATGGGAAACTATC
SkWmt-R1	TTATGGCATGCTATTGTCCC
SkWmt-R2	GTCATACCAGCTAATCAAGC
SkW-F1	CTTATTTGCTGAGATGTCCC
SkW-F2	GTGATTCAGACAGCTTATCC
SkW-R	CTAGTCGCAATAAGTGATGC

Table S3. Primer binding sites coordinates in sequence files.

target	Primer	SK1-start	SK1-end	W303-start	W303-end
1 chr	SkW-F1	177974	177993	181764	181783
	SkW-F2	162597	162616	160456	160475
	SkW-R	178902	178883	162076	162057
mtDNA	SkWmt-F	74850	74869	12582	12601
	SkWmt-R1	72608	72589	12893	12874
	SkWmt-R2	75505	75486	15756	15737

SK1 sequence: https://www.ncbi.nlm.nih.gov/datasets/genome/GCA_002057885.1/

W303 sequence: https://www.ncbi.nlm.nih.gov/datasets/genome/GCA_002163515.1/

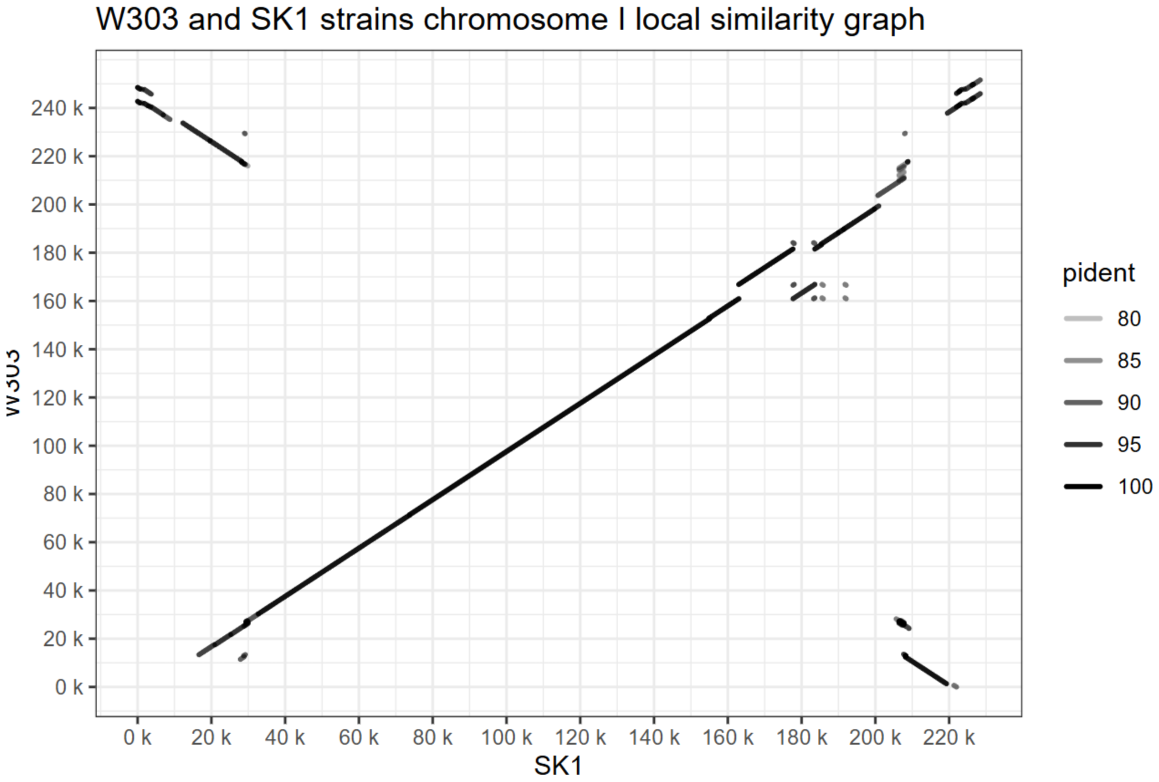

Figure S1. Local similarity graph for chromosome 1 of *SK1* and *W303* strains. The rearrangement used for verifying PCR is shown at approximately 160,000 base pairs (160 kb) along the genomic coordinates.

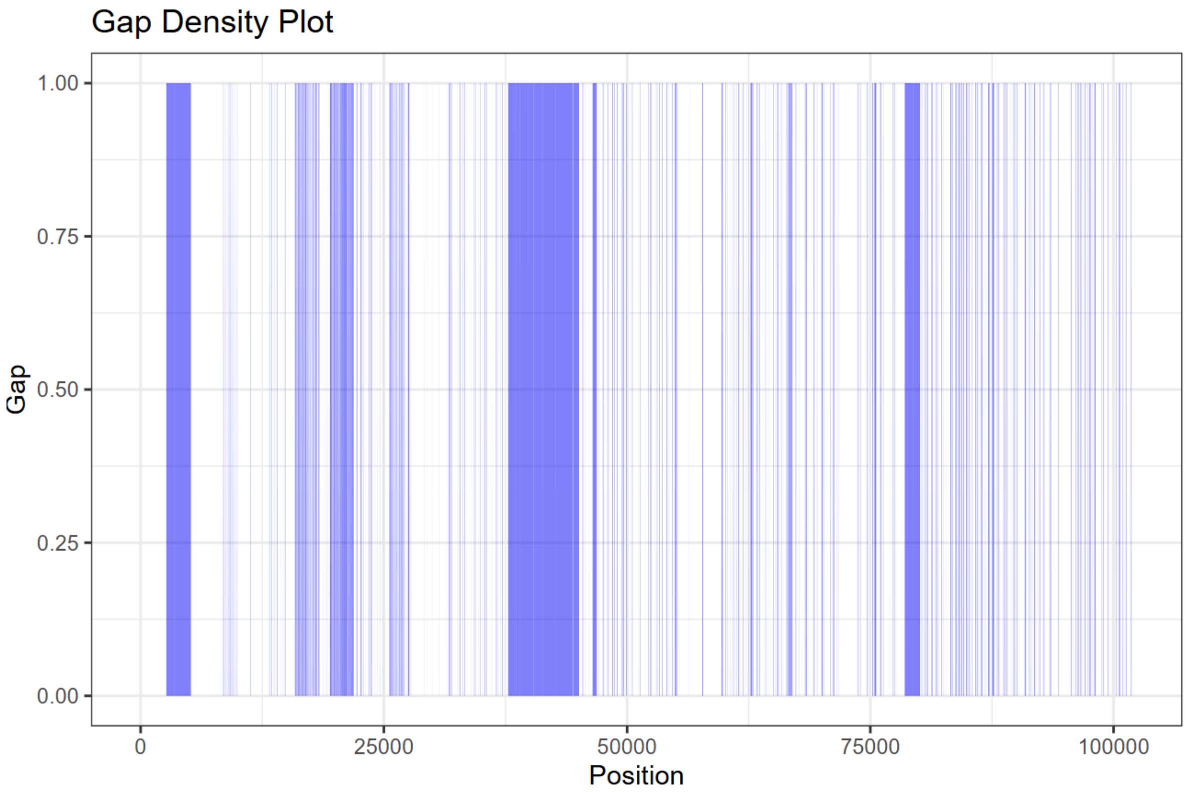

Figure S2. Gaps in the alignment of *SK1* and *W303* mitochondrial DNA. This figure shows the gaps in the alignment of *SK1* and *W303* mtDNA that were excluded from the analysis presented in Figure 1B. Blue bands represent columns with gaps. Both sequences start at the *CO1* gene.

Supplementary Text S1. Detecting missense mutations in *PET127*, *CCE1* and *RPO41*

In order to characterize *SK1* and *W303* strains differences in genes *PET127*, *CCE1* and *RPO41*, that are known to influence suppressivity, we utilized the tool https://www.yeastgenome.org/strainAlignment. It allows to align protein sequences for each gene in *S. cerevisiae* popular strains. This revealed 2-4 missense mutations between the *SK1* and *W303* strains in the aforementioned genes. Using the “quick blastp” programme [https://blast.ncbi.nlm.nih.gov/Blast.cgi?PROGRAM=blastp] within *Ascomycota* and not *S. cerevisiae* proteins in non-redundant protein banks we found hits for each protein with identity of more than 50%. We then performed multiple alignments of these hits with the protein sequences from *SK1*, *W303* and *S288C* in JalView [https://www.jalview.org/development/archive/Version-2_11_4_0/] using Muscle with default parameters. All identified mutations are located in non-conserved columns, and do not involve unusual for this position residues.

Conde, J., and G. R. Fink. 1976. “A Mutant of Saccharomyces Cerevisiae Defective for Nuclear Fusion.” *Proceedings of the National Academy of Sciences of the United States of America* 73 (10): 3651–55.

Karavaeva, I.E.; Golyshev, S.A.; Smirnova, E.A.; Sokolov, S.S.; Severin, F.F.; Knorre, D.A. Mitochondrial Depolarization in Yeast Zygotes Inhibits Clonal Expansion of Selfish mtDNA. *J. Cell Sci.* 2017, *130*, 1274–1284.

Kashko, Nataliia D., Iuliia Karavaeva, Elena S. Glagoleva, Maria D. Logacheva, Sofya K. Garushyants, and Dmitry A. Knorre. 2024. “Inheritance Bias of Deletion-Harbouring mtDNA in Yeast: The Role of Copy Number and Intracellular Selection.” *bioRxiv*. https://doi.org/10.1101/2024.09.11.612442.
